# The airway microbiome of persons with cystic fibrosis correlates with acquisition and microbiological outcomes of incident *Stenotrophomonas maltophilia* infection

**DOI:** 10.3389/fmicb.2024.1353145

**Published:** 2024-04-16

**Authors:** Lauren A. Bowron, Nicole Acosta, Christina S. Thornton, Jennifer Carpentero, Barbara-Jean M. Waddell, Lalit Bharadwaj, Kirsten Ebbert, Daniel Castañeda-Mogollón, John M. Conly, Harvey R. Rabin, Michael G. Surette, Michael D. Parkins

**Affiliations:** ^1^Department of Microbiology, Immunology and Infectious Diseases, University of Calgary, Calgary, AB, Canada; ^2^Department of Medicine, University of Calgary, Calgary, AB, Canada; ^3^Department of Pediatrics, University of Calgary, Calgary, AB, Canada; ^4^Department of Microbiology, McMaster University, Hamilton, ON, Canada

**Keywords:** microbiome, cystic fibrosis, bronchiectasis, emerging infections, *Stenotrophomonas maltophilia*, incident

## Abstract

**Rationale:**

Chronic infection with *Stenotrophomonas maltophilia* in persons with cystic fibrosis (pwCF) has been linked to an increased risk of pulmonary exacerbations and lung function decline. We sought to establish whether baseline sputum microbiome associates with risk of *S. maltophilia* incident infection and persistence in pwCF.

**Methods:**

pwCF experiencing incident *S. maltophilia* infections attending the Calgary Adult CF Clinic from 2010–2018 were compared with *S. maltophilia*-negative sex, age (+/−2 years), and birth-cohort-matched controls. Infection outcomes were classified as persistent (when the pathogen was recovered in ≥50% of cultures in the subsequent year) or transient. We assessed microbial communities from prospectively biobanked sputum using V3-V4 16S ribosomal RNA (rRNA) gene sequencing, in the year preceding (Pre) (*n* = 57), at (At) (*n* = 22), and after (Post) (*n* = 31) incident infection. We verified relative abundance data using *S. maltophilia*-specific qPCR and 16S rRNA-targeted qPCR to assess bioburden. Strains were typed using pulse-field gel electrophoresis.

**Results:**

Twenty-five pwCF with incident *S. maltophilia* (56% female, median 29 years, median FEV_1_ 61%) with 33 total episodes were compared with 56 uninfected pwCF controls. Demographics and clinical characteristics were similar between cohorts. Among those with incident *S. maltophilia* infection, sputum communities did not cluster based on infection timeline (Pre, At, Post). Communities differed between the infection cohort and controls (*n* = 56) based on Shannon Diversity Index (SDI, *p* = 0.04) and clustered based on Aitchison distance (PERMANOVA, *p* = 0.01) prior to infection. At the time of incident *S. maltophilia* isolation, communities did not differ in SDI but clustered based on Aitchison distance (PERMANOVA, *p* = 0.03) in those that ultimately developed persistent infection versus those that were transient. *S. maltophilia* abundance within sputum was increased in samples from patients (Pre) relative to controls, measuring both relative (*p* = 0.004) and absolute (*p* = 0.001). Furthermore, *S. maltophilia* abundance was increased in sputum at incident infection in those who ultimately developed persistent infection relative to those with transient infection, measured relatively (*p* = 0.04) or absolute (*p* = 0.04), respectively.

**Conclusion:**

Microbial community composition of CF sputum associates with *S. maltophilia* infection acquisition as well as infection outcome. Our study suggests sputum microbiome may serve as a surrogate for identifying infection risk and persistence risk.

## 1 Introduction

Cystic fibrosis (CF) is characterized by repeated cycles of infection and inflammation resulting from dysfunctional CF transmembrane conductance regulators and the accumulation of thick and sticky mucus lining the airways (Fahy and Dickey, 2010). Repeated and chronic airway infections result in airway remodeling, bronchiectasis and ultimately respiratory failure – the leading cause of death in persons with CF (pwCF) (Goetz and Ren, 2019). Bacterial pathogens are the primary drivers of these repeated airway infections (De Vrankrijker et al., 2010); (LiPuma, 2010). Recently, culture-independent methods have revealed that the airway is host to a diverse community of microorganisms – many not identified through standard clinical cultivation techniques (Dickson et al., 2020). We have previously demonstrated that the CF sputum microbiome may serve as a biomarker in predicting long-term outcomes (Acosta et al., 2018) and treatment response (Heirali et al., 2017, 2019, 2020).

*Stenotrophomonas maltophilia* is an obligate aerobic Gram-negative capable of invading and causing chronic infection within the nutrient-deficient CF airway (Calza et al., 2003; Brooke, 2012). The prevalence of *S. maltophilia* infection in CF has increased from 3% in 1999 to ~15% in recent years (Marchac et al., 2004; Capaldo et al., 2020). *S. maltophilia* is intrinsically multi-drug resistant, which presents challenges in treatment (Gibb and Wong, 2021). Chronic infection is associated with an increased risk of poor outcomes including pulmonary exacerbation (PEx), lung function decline, and progression to end-stage lung disease manifesting as need for life saving lung transplantation or resulting in death (Waters et al., 2011, 2012; Amin et al., 2020). A recent retrospective cross-sectional study demonstrated that incident infection acquisition with *S. maltophilia* was associated with a reduction in lung function (Barsky et al., 2017). Despite observed negative clinical outcomes, there is a paucity of data around incident infections of *S. maltophilia* and currently represents a knowledge gap. We thus sought to determine if baseline sputum microbiota associates with susceptibility to incident airways infection and microbiological outcome with *S. maltophilia.* To our knowledge, this is the first study to investigate the CF airway microbiome in the context of incident infections with *S. maltophilia* exploring an association of the CF airway microbiome with infection acquisition and persistence risk.

## 2 Methods

### 2.1 Participant identification and sample collection

PwCF attending the Calgary Adult Cystic Fibrosis Clinic (CACFC) are seen quarterly and during periods of PEx at which times they contribute to the CACFC Sputum Biobank – a prospective repository of over 22,000 sputum samples linked to clinical outcomes, approved by the regional ethics board (REB15-0854). In real-time, sputum samples were processed by the clinical laboratory following established procedures to identify CF pathogens. Sputum-derived pathogens were prospectively frozen in glycerol and transferred to the CACFC Strain Biobank. All sputum samples were maintained at −80°C for future analysis. These sampling procedures were consistent with previous sputum microbiome studies (Acosta et al., 2017, 2018; Heirali et al., 2017, 2019, 2020; Thornton et al., 2022).

Participants were identified based on a clinical history of *S. maltophilia* infection. Inclusion criteria for participants with *S. maltophilia* infection were: adults aged ≥18 years with a diagnosis of CF, the occurrence of incident infection while attending the CACFC between 2010 and 2018, and the availability of at least one sputum sample in the biobank from the year before/at the first identification of *S. maltophilia*. Participants were excluded if they had a history of *S. maltophilia* infection in the 5 years preceding the collection date and were only included after the 5-year mark if strain typing confirmed the occurrence of a new strain. Consistent with a previous study by our group (Acosta et al., 2017), two samples in the year before infection (Pre), the sample at incident infection (when a new strain of S. maltophilia was first identified) (At) and a sample in the year after incident infection (Post) were assessed, where available. A control cohort was identified contingent on not having had *S. maltophilia* identified in their sputum, including a period of at least 2 years of observed culture-free status. Controls were matched 2:1 based on age (±2 years) and sex. Two sputum samples from each control were used if they were collected within 2 years to the date of each corresponding incident infection case. Any sputum sample that had been collected within 4 weeks of a change in antimicrobial therapy, or systemic antibiotics were not considered for the study on the basis of potential confounding effects (Dickson and Morris, 2017; Noci et al., 2018).

### 2.2 Clinical outcomes definitions

Incident infection was defined as presence of an *S. maltophilia* strain that had not previously been identified in each pwCF as determined by pulse field gel electrophoresis (PFGE). For purposes of this study infection and colonization are not differentiated, and the descriptor “infection” used throughout the manuscript relates to the recovery of *S. maltophilia* from CF respiratory secretions. Infections were classified as either transient or persistent. Persistence was defined when cases had at least half of their cultures (rounded up) positive for *S. maltophilia* in the year following the incident date and a minimum of 3 sputum samples in that year.

### 2.3 DNA extraction

Total genomic DNA was isolated from sputum following protocols as previously described (Acosta et al., 2017) and described in the Supplemental File.

### 2.4 16S rRNA amplification and sequencing

A modified protocol (see the Supplemental File) of Bartram et al. (2011) was used to amplify the V3-V4 region of the 16S rRNA gene with 3VF and V4Rmod2 (Integrated DNA Technologies, Coralville, Iowa, USA) primers using 96 unique barcoded primers for paired-end Illumina MiSeq sequencing (Illumina, Inc., San Diego, USA [RRID: SCR_016379]) (Caporaso et al., 2011; Whelan et al., 2014). Amplified DNA samples were sent to the McMaster Genome Facility (Hamilton, Ontario) for sequencing (Whelan et al., 2014; Lam et al., 2015). The raw paired-end FASTQ files generated from sequencing were then processed for microbial community analysis.

### 2.5 Microbial communities analysis

Sequence processing and downstream analysis were performed in R Studio (RRID: SCR_000432) (v. 1.4), R (v. 4.1.1). In brief, the barcoded primers and adaptor sequences were removed using Cutadapt (RRID: SCR_011841) (v. 1.2.1) (Martin, 2011), followed by filtering trimming, sample inference, alignment, and finally, taxonomy assignment using the Divisive Amplicon Denoising Algorithm 2 (DADA2 [RRID: SCR_023519]) (Callahan et al., 2016).

A comparison of the taxonomic composition through the natural history of infection was assessed at the level of the 15 most prevalent ASVs to achieve <95% of read coverage (Nearing et al., 2022). As the distribution of read counts per amplicon were observed to be non-normally distributed, we conducted a chi-square goodness of fit test. To assess community composition, α-diversity was calculated using the Shannon Diversity Index (SDI) and Observed Diversity Index (ODI). The SDI and ODI were considered because they have been previously established measures that effectively assessed CF sputum community evenness and richness (Jost, 2006).

Principal Component Analysis (PCA) using CLR (Center-log ratio) transformation was used to determine the dissimilarity between samples as represented by the β-diversity in the microbiome (Gloor et al., 2017). To assess the dissimilarity across the natural history of infection, PCA was performed on Pre, At, and Post samples in the *S. maltophilia* cohort. PCA was also conducted to compare the pre-infection and uninfected control samples and those with persistent versus transient infection. Permutational multivariate analysis of variance (PERMANOVA) using the Aitchison distance with the adonis function of the vegan package (Okasanen et al., 2022) was performed to investigate the significance of the factors that contributed to any observed differences (Garcia-Nuñez et al., 2020). PCA was selected as it is a commonly used visualization tool that can summarize variance among the data (Paliy and Shankar, 2016). CLR transformation was conducted on account of the compositional nature of the data and served to capture the relationships between the features in the data. Thus we can compare the abundances of features relative to other features (Gloor et al., 2017). This transformation has been shown to be suitable for multivariate analyses such as PCA (Paliy and Shankar, 2016). The top 6 taxa influencing the PCA dissimilarity were plotted on the PCA plot. Taxa were filtered with a minimum prevalence of a proportion of 10% of all samples/reads to reduce the sensitivity to extremely rare taxa (Cao et al., 2020). Permutational analysis was conducted using distance matrices with 1,000 permutations on normalized data using the CLR method (Anderson, 2001). The R package, DESeq2 (RRID: SCR_015687) was used for differential abundance analysis (DAA). DESeq2 was selected since it uses a negative binomial distribution, which is closely related to the observed Poisson Lognormal distribution of our data (Nearing et al., 2022). DAA was used to identify differences in the relative abundance of taxa among the Pre samples compared with the uninfected controls and between the persistent infection samples and the transient infection samples. Taxa were excluded if they were not observed in more than one sample (prevalence ≥2).

### 2.6 Strain-level identification

Pulse-field gel electrophoresis (PFGE) was used to identify incident strains of *S. maltophilia* and identify episodes of infection following previously published protocols (Parkins et al., 2014) and modified from Aaron et al. (2010) – full details in Supplementary File. PFGE was conducted for the 6 participants that had multiple episodes of *S. maltophilia* infection collected.

### 2.7 Absolute quantification of DNA

Quantitative PCR (qPCR) using TaqMan Fast Advanced was used to confirm the presence and absolute abundance of *S. maltophilia* (Fraser et al., 2019) and total-bacterial 16S rRNA (Cho et al., 2021) following adapted protocols (full details in the Supplementary File).

### 2.8 Statistical analysis

Non-parametric Wilcoxon rank-sum tests or two-tailed Fisher exact probability tests were performed on the comparisons of participant demographics and clinical characteristics (Heirali et al., 2017, 2020; Breen et al., 2022). A non-parametric Kruskal-Wallis test followed by the Dunn’s Test with the Bonferroni method to correct for multiple comparisons was used to compare the α-diversity of the natural history of *S. maltophilia* infection among the Pre, At, and Post samples (Heirali et al., 2017). Wilcoxon rank-sum tests were used to assess the differences in α-diversity. Additionally, Wilcoxon rank sum tests were used to compare the differences in abundance, both relative and absolute abundance between the case and control samples, as well as between the persistent and transient infection samples (Caverly et al., 2019; Heirali et al., 2020). All analyses were performed in R Studio (v. 1.41717), R (v. 4.1.1).

## 3 Results

### 3.1 Participant demographics

Twenty-five pwCF with 33 incident *S. maltophilia* infections met the inclusion and exclusion criteria (14 females; 11 males, median age 29.0 years (interquartile range [IQR] 24.1–39.6), median ppFEV_1_ 61.0 (IQR 48.0–82.0). In the cohort, 19 pwCF (76%) had only one episode of *S. maltophilia* infection, 4 pwCF (16%) had two episodes (median time between episodes 2.1 years [IQR 1.8–3.0]), and 2 pwCF (8%) had three episodes (median time between episodes 1.7 years [IQR 1.4–1.7]). From the cohort, 57 pre-infection samples were collected (median 5.1 months (IQR 2.7–6.9 months) from the date of incident *S. maltophilia* infection), 22 at-infection samples, and 31 post-infection samples (median 3.0 months [IQR 2.0–5.0 months]). In pwCF with first incident infection, median ppFEV_1_, as surrogate for disease severity, did not differ between Pre (60.0 [IQR 46.0–78.0]), At [67.0 (IQR 50.0–85.5)], and Post (67.0 [IQR 49.0–74.3]) *p* = 0.658). Of the 33 incident infection events, 20 (60.6%) included all three time points; at least one pre-infection, at-infection, and post-infection, 9 (27.3%) included only pre-infection and post-infection, 2 (6.1%) included only at-infection and post-infection, and 2 (6.1%) included only pre-infection.

Of the 33 *S. maltophilia* incident infection events, 18 (54.5%) were defined as persistent infections. Transient and persistent infections among first episode cases were similar across demographic and clinical characteristics including age (Wilcoxon; *p* = 0.851), sex (Wilcoxon; *p* = 0.089), lung function as measured by ppFEV_1_ (Wilcoxon; *p* = 0.328) and ppFVC (Wilcoxon; *p* = 0.103), BMI (Wilcoxon; *p* = 0.336), proportion homozygous for F508del (Fisher’s exact test; *p* = 0.102), and CF-related comorbidities (*p* > 0.05) (Table 1). There were no differences in trimethoprim-sulfamethoxazole susceptibilities detected between transient and persistent samples (*p* = 1) (Supplementary Table S1).

**Table 1:** Characteristics of cases that develop transient and persistent *S. maltophilia* infection prior to infection recorded at the first episode pre-infection time point.

Demographics		
	Transient (*n* = 14)	Persistent (*n* = 11)
Female Sex	7 (50.0%)	7 (63.6%)
Age At Infection (years)	32.0 (24.3–39.1)	28.2 (25.1–36.2)
ppFEV_1_	62.0 (57.0–82.0)*	56.0 (42.5–71.3)**
ppFVC	90.0 (58.0–103.0)*	66.5 (55.8–85.3)**
BMI	21.6 (19.9–23.2)*	20.3 (19.1–21.6)**
F508del/F508del	1 (7.1%)	6 (54.5%)
F508del/Other	4 (28.6%)	2 (18.2%)
Other/Other	1 (7.1%)	0 (0%)
Unknown	8 (57.1%)	3 (27.3%)
CF-related Comorbidities		
	**Transient (***n* **= 9)**	**Persistent (***n* **= 8)**
Pancreatic Insufficiency	8 (88.8%)	8 (100.0%)
CF-Related Diabetes	4 (44.4%)	2 (25.0%)
CF Liver Disease	3 (33.3%)	4 (50.0%)
Distal Intestinal Obstructive Syndrome	3 (33.3%)	1 (12.5%)
Osteopenia/Osteoporosis	5 (55.5%)	5 (62.5%)
Sinus Disease	7 (77.7%)	6 (75.0%)

CF-related comorbidity data was unavailable for 8 cases (5 transient cases and 3 persistent cases). Data are presented as either median (interquartile range) or N (%). Either Wilcoxon rank-sum test or Fisher-exact probability at two-tailed were performed (*p* > 0.05).

ppFEV1, percent predicted forced expiratory volume in 1 second; ppFVC, percent predicted forced vital capacity; BMI, Body mass index.

*Denotes an *n* of 13 due to unavailability of clinical data for 1 case.

**Denotes an *n* of 8 due to unavailability of clinical data for 3 cases.

Fifty-six *S. maltophilia* uninfected controls were identified (20 pwCF were matched with two controls and 5 pwCF were matched with one control). Where only 1 control was identified it related to the inability to match on basis of age/sex – generally due to outlier status.

Case and control samples measured at the first infection episode (*n* = 44) did not differ based on lung function as measured by either ppFEV_1_ (Wilcoxon; *p* = 0.371) or ppFVC (Wilcoxon; *p* = 0.103), BMI (Wilcoxon; *p* = 0.221), or on whether they were homozygous for F508del (Fisher’s exact test; *p* = 1.00). Case and controls were similar across CF-related co-morbidities (*p* > 0.05) (Table 2).

**Table 2:** Cohort characteristics and CF-related comorbidities of those with *S. maltophilia* incident infection and controls recorded at the first episode pre-infection time point.

Demographics		
	Control (*n* = 44)	Case (*n* = 25)
Age At Infection (years)	28.9 (22.7–38.1)	29.0 (24.1–39.6)
ppFEV_1_	73.0 (50.0–92.5)*	61.0 (48.0–82.0)**
ppFVC	95.0 (71.5–102.5)*	86.0 (56.0–96.0)**
BMI (kg/m^2^)	22.0 (19.8–24.2)*	20.8 (19.5–22.2)**
F508del/F508del	20 (45.5%)	7 (28.0%)
F508del/Other	13 (29.5%)	6 (24.0%)
Other/Other	9 (20.5%)	1 (4.0%)
Unknown	2 (4.5%)	11 (44.0%)
CF-related Comorbidities		
	**Control (*n* = 43)**	**Case (*n* = 17)**
Pancreatic Insufficiency	38 (88.4%)	16 (94.1%)
CF-Related Diabetes	16 (37.2%)	6 (35.3%)
CF Liver Disease	7 (16.3%)	7 (41.2%)
Distal Intestinal Obstructive Syndrome	8 (18.6%)	4 (23.5%)
Osteopenia/Osteoporosis	19 (44.2%)	10 (58.8%)
Sinus Disease	30 (69.8%)	13 (76.5%)

CF-related comorbidity data were unavailable for 8 cases and unavailable for 1 control.

Data are presented as either median (interquartile range) or N (%).

Either Wilcoxon rank-sum test or Fisher-exact probability at two-tailed were performed (*p* > 0.05).

ppFEV1, percent predicted forced expiratory volume in 1 second; ppFVC, percent predicted forced vital capacity; BMI, Body mass index.

*Denotes an *n* of 43 due to unavailability of clinical data for 1 control.

**Denotes an *n* of 21 due to unavailability of clinical data for 4 cases.

### 3.2 Cystic fibrosis sputum microbiome composition

**Figure 1:**
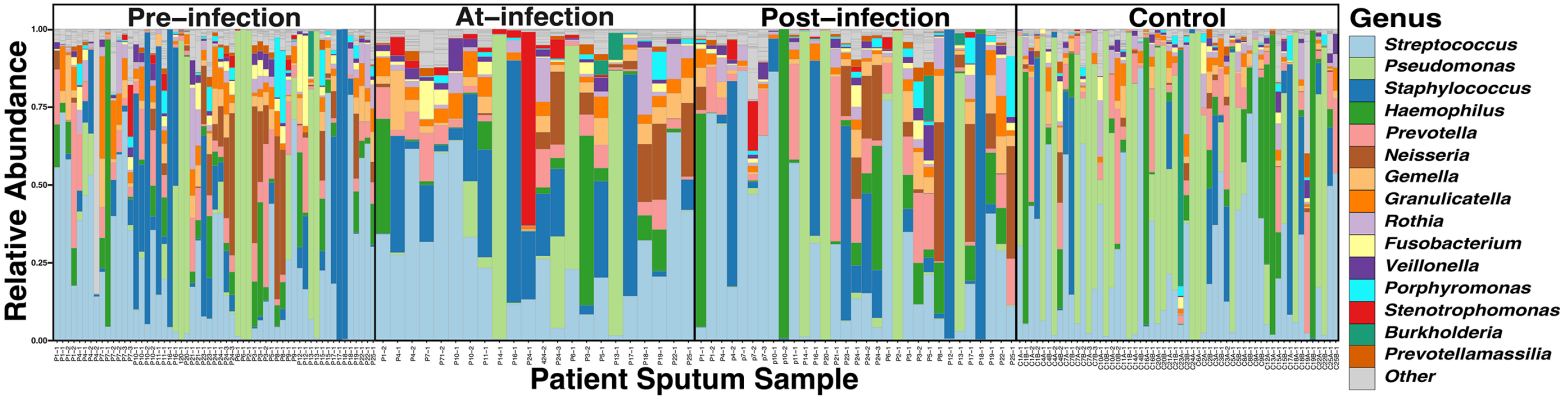
Comparison of the taxonomic composition of the top 15 ASVs in CF sputum through the course of incident *S. maltophilia* infection (*n* = 25) and uninfected controls (*n* = 56). Relative abundance was collected at the genus level for samples at pre-infection (samples = 57; median 5.1 months from incident infection date [interquartile range (IQR) 2.7–6.9 months)], at-infection (samples = 22), and post-infection (samples = 31; median 3.0 months from incident infection date [IQR 2.0–5.0 months)], and control samples corresponding to case infection dates.

Across the 166 sputum samples assessed, a total of 6,713,780 reads (69,935.21 reads/sample, IQR, 40,728 – 95,364) with a total of 1,261 Amplicon Sequence Variants (ASVs) were identified. To assess the quality of the 16S sequencing, a species accumulation curve (SAC) analysis was performed (Supplementary Figure S1). The comparison of taxonomic composition through the natural history of infection showed the relative abundance of the 15 most prevalent ASVs accounted for 95.7% of the reads (Figure 1). All of the ASVs corresponded to taxa that have been previously identified as CF airway genus-level constituents: *Streptococcus* (27.8%), *Pseudomonas* (15.8%), *Staphylococcus* (14.8%), *Haemophilus* (7.8%), *Prevotella* (6.1%), *Neisseria* (5.7%), *Gemella* (3.8%), *Granulicatella* (3.6%), *Rothia* (2.0%)*, Porphyromonas* (1.6%), *Fusobacterium* (1.5%)*, Stenotrophomonas* (1.4%), *Veillonella* (1.3%), *Sneathia*, (1.0%), *Prevotellamassilia* (0.6%)(Stressmann et al., 2012; Surette, 2014). The distribution of the read counts per amplicon across samples were found to follow a Poisson-Lognormal distribution (*p* < 0.0001).

### 3.3 Sputum community dynamics through *Stenotrophomonas maltophilia* incident infection

**Figure 2:**
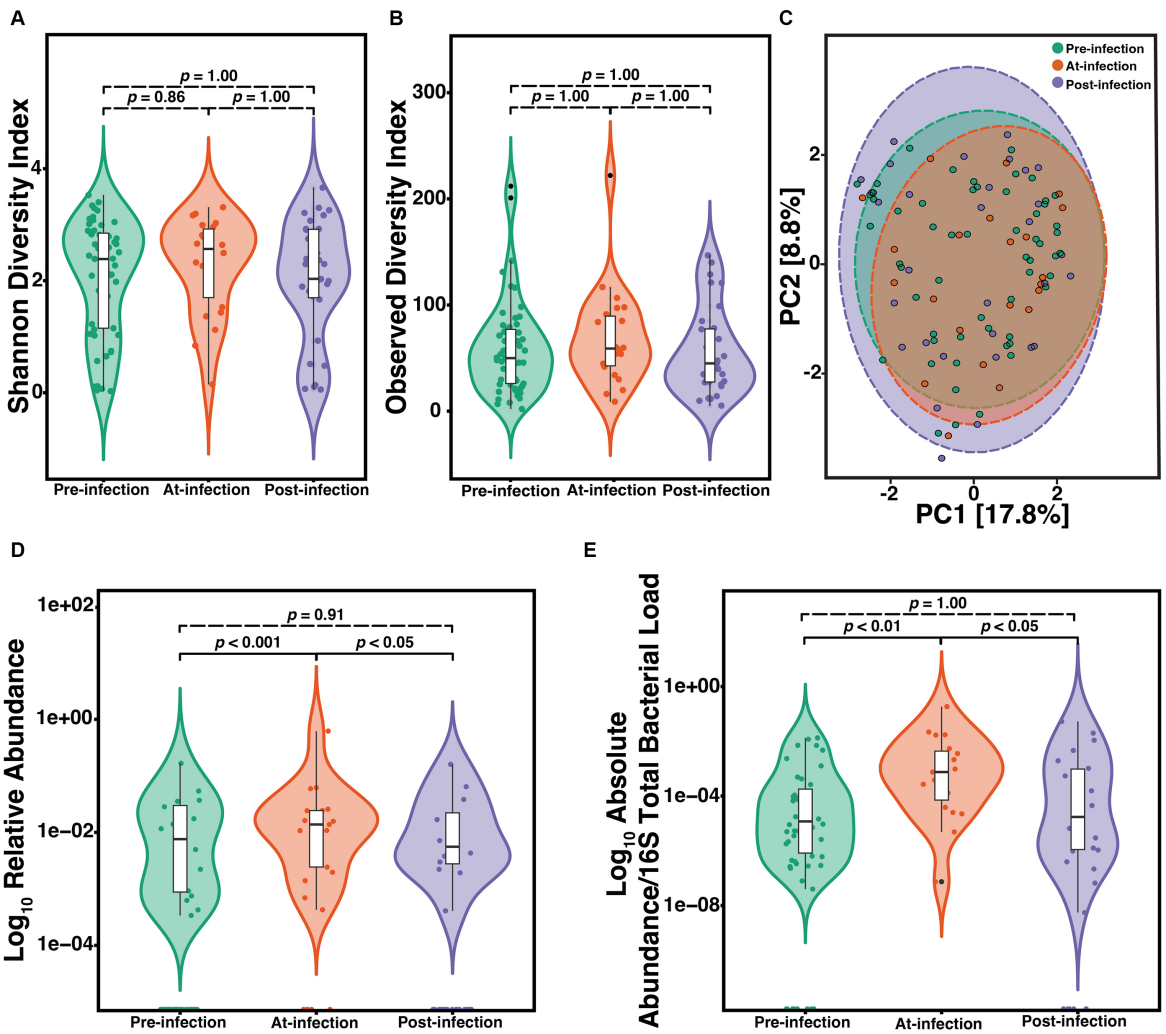
Natural history of the CF lung microbiome through course of incident *S. maltophilia* infection. Samples included are case samples at 6-months to one-year pre-infection (*n* = 57), at-infection (*n* = 22), and within 1-year post-infection (*n* = 31). **(A)** Shannon diversity index (SDI) (Kruskal-Wallis, *p* = 0.63). **(B)** Observed diversity index (ODI) (Kruskal-Wallis, *p* = 0.42) **(C)** Principal component analysis with center-log ratio (CLR) transformation; (PERMANOVA, *p* = 0.991). **(D)** Relative abundance of *S. maltophilia* for pre-infection, at-infection, and post-infection presented in the Log10 scale (Kruskal-Wallis, *p* = 0.00029). Significant differences were detected between pre-infection and at-infection (Wilcoxon, *p* = 6.6e-05) and at-infection and post-infection (Wilcoxon, *p* = 0.0094). **(E)** Absolute abundance/total 16S bacterial load for pre-infection, at-infection, and post-infection (Kruskal-Wallis, *p* = 0.00089). Significant differences in abundance were detected between pre-infection and at-infection (Wilcoxon, *p* = 4.1e-05) and between at-infection and post-infection (Wilcoxon, *p* = 4.1e-05) presented in the Log10 scale. Data points are represented as colored dots. Outlier data points are black and are defined as any point that were beyond 1.5 times the interquartile range.

To determine the natural history of community composition through *S. maltophilia* infection, we compared the α-diversity of the microbiome between sputum Pre (*n* = 57), At (*n* = 22), and Post (*n* = 31) incident infection. We did not find any significant differences among Pre, At, and Post samples as measured by either the SDI (Kruskal-Wallis, *p* = 0.63) or the ODI (Kruskal-Wallis, *p* = 0.42) (Figures 2A,B). Similar results were observed when limiting the analysis to just the first episode of infection and when only considering the first pre-infection sample (data not shown). Principal component analysis (PCA) was used to visualize any potential clustering patterns among the sputum samples corresponding to the infection stage (Figure 2C). Samples did not cluster by infection stage (PERMANOVA, *p* = 0.991). The strongest driver of microbial community structure was participant ID, the unique identifier given to each participant (PERMANOVA; *F* = 3.9, R^2^ = 52.2%, *p* = 0.001). Considering the strong contribution of participant ID to community structure, for each subsequent analysis we additionally blocked the metadata by the participant and then used PERMANOVA to support each analysis which showed similar results. We assessed the difference in relative abundance of *S. maltophilia* at Pre, At, and Post and found significant differences between Pre and At (*p* = 6.6e-05) and At and Post (*p* = 0.0094) (Figure 2D). These differences were also observed when comparing the ratio of the absolute abundance of *S. maltophilia* to absolute abundance of 16S rRNA total bacterial burden between Pre and At (*p* = 0.00016) and between At and Post (*p* = 0.006) (Figure 2E). Similar results were observed when limiting the analysis to only the first episode of infection and when only considering the first pre-infection sample (data not shown).

### 3.4 Differences in the microbiome among pwCF who acquire *Stenotrophomonas maltophilia* infection

**Figure 3:**
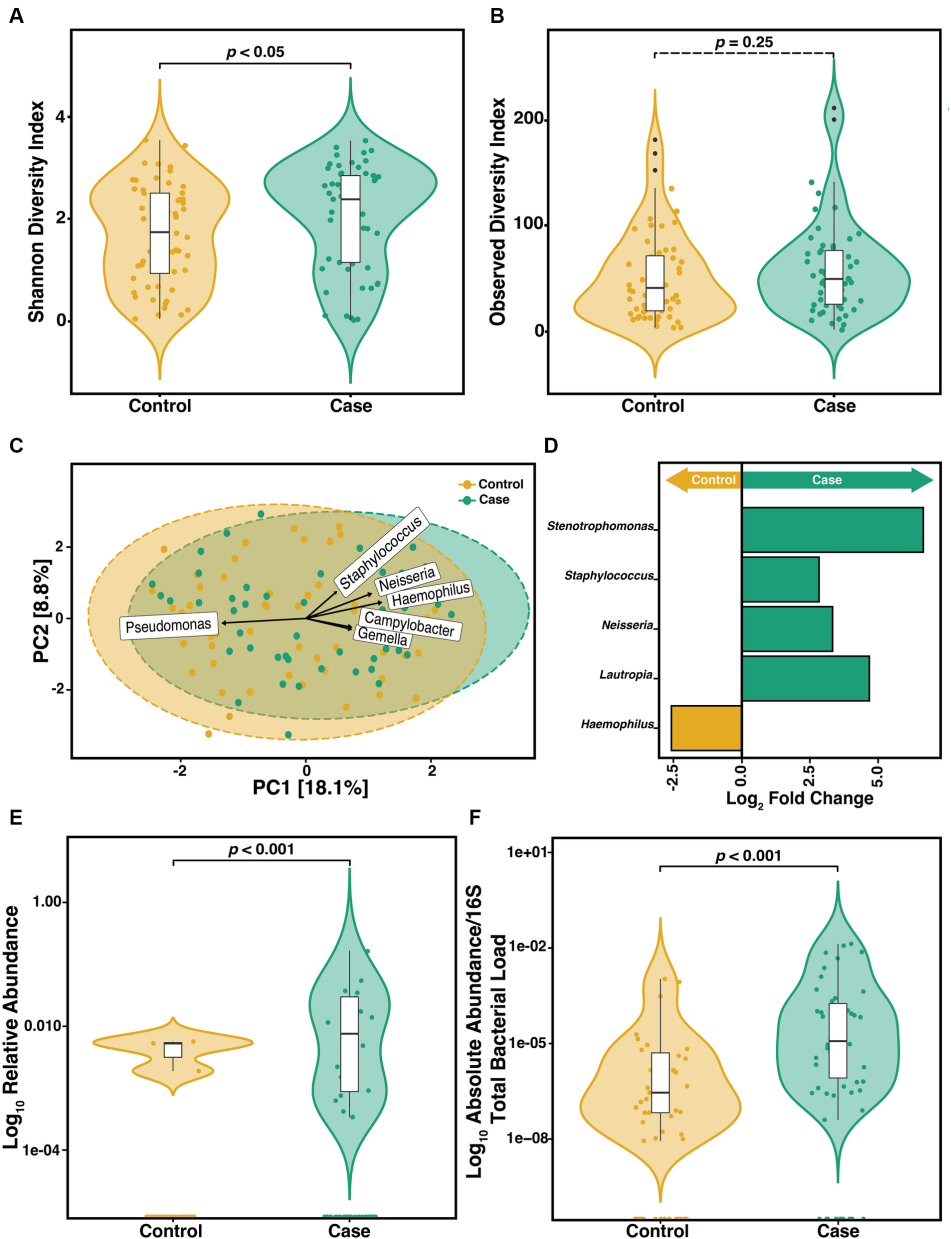
Microbiome diversity between pre-infection (*n* = 57) *S. maltophilia* sputum samples and uninfected controls (*n* = 56). **(A)** Shannon Diversity Index (SDI) (Wilcoxon, *p* = 0.04) **(B)** Observed Diversity Index (ODI) (Wilcoxon, *p* = 0.25). **(C)** Principal Component Analysis (PCA) with center-log ratio (CLR) transformation; (PERMANOVA, *p* = 0.01) with top 6 taxa, present in ≥10% of samples, influencing unconstrained PCA clustering. **(D)** Taxa with significant log fold changes in relative abundance between pre-infection samples and uninfected controls, detected by DESeq2 (Wald test; *p*-adjusted<0.05). **(E)** Relative abundance of *S. maltophilia* for control and pre-infection case samples presented in the Log10 scale (Wilcoxon *p* = 0.0013). **(F)** Absolute abundance/total 16S bacterial load for controls (*n* = 49) and pre-infection case samples (n = 50) presented in the Log10 scale (Wilcoxon, *p* = 4.1e-05). Data points are represented as colored dots. Outlier data points are black and are defined as any point that were beyond 1.5 times the interquartile range.

To determine if the constituents of the CF sputum microbiome differ between pwCF that acquire *S. maltophilia* infection and uninfected controls, we compared the α-diversity of sputum in the year preceding incident infection. We observed a difference in the SDI (Wilcoxon, *p* = 0.04) (Figure 3A) but not ODI (Wilcoxon, *p* = 0.25) (Figure 3B). Beta-diversity ordination was calculated by using PCA to determine clustering patterns between pre-infection and uninfected controls (Figure 3C). We found that community structure clustered based on the cohort (PERMANOVA, *p* = 0.01). Similar results were observed when data were stratified by participant ID (PERMANOVA, *p* = 0.001). We identified the top 6 ASVs that contributed to this dissimilarity in clustering and conducted DAA with DESeq2 to identify the ASVs that differed (*p*-adjusted<0.05) between groups. We found that *Haemophilus*, *Neisseria*, *Campylobacter, Gemella,* and *Staphylococcus* contributed to the difference in clustering in the pre-infection samples whereas *Pseudomonas* was a contributor to the clustering difference in the control samples (Figure 3C). *Neisseria* (*p* = 0.01), *Staphylococcus* (*p* = 0.02), *Lautropia* (*p* = 0.02), and *Stenotrophomons* (*p* = 0.002) genera were significantly enriched, and *Haemophilus* (*p* = 0.02) were diminished in cases versus controls (Figure 3D). Despite sputum samples collected before infection identification (Pre) being culture negative for *S. maltophilia*, we found its relative abundance to be increased compared to uninfected controls (Figure 3E) (Wilcoxon, *p* = 0.0013). To support this observation, we confirmed the absolute abundance of *S. maltophilia* total 16S both at pre-infection and at-infection by qPCR (Figure 3F) (Wilcoxon, *p* = 4.1e-05). Similar results were obtained when assessing only the first episode from each case.

### 3.5 Microbiome structure at incident acquisition as a function of infection outcome

**Figure 4:**
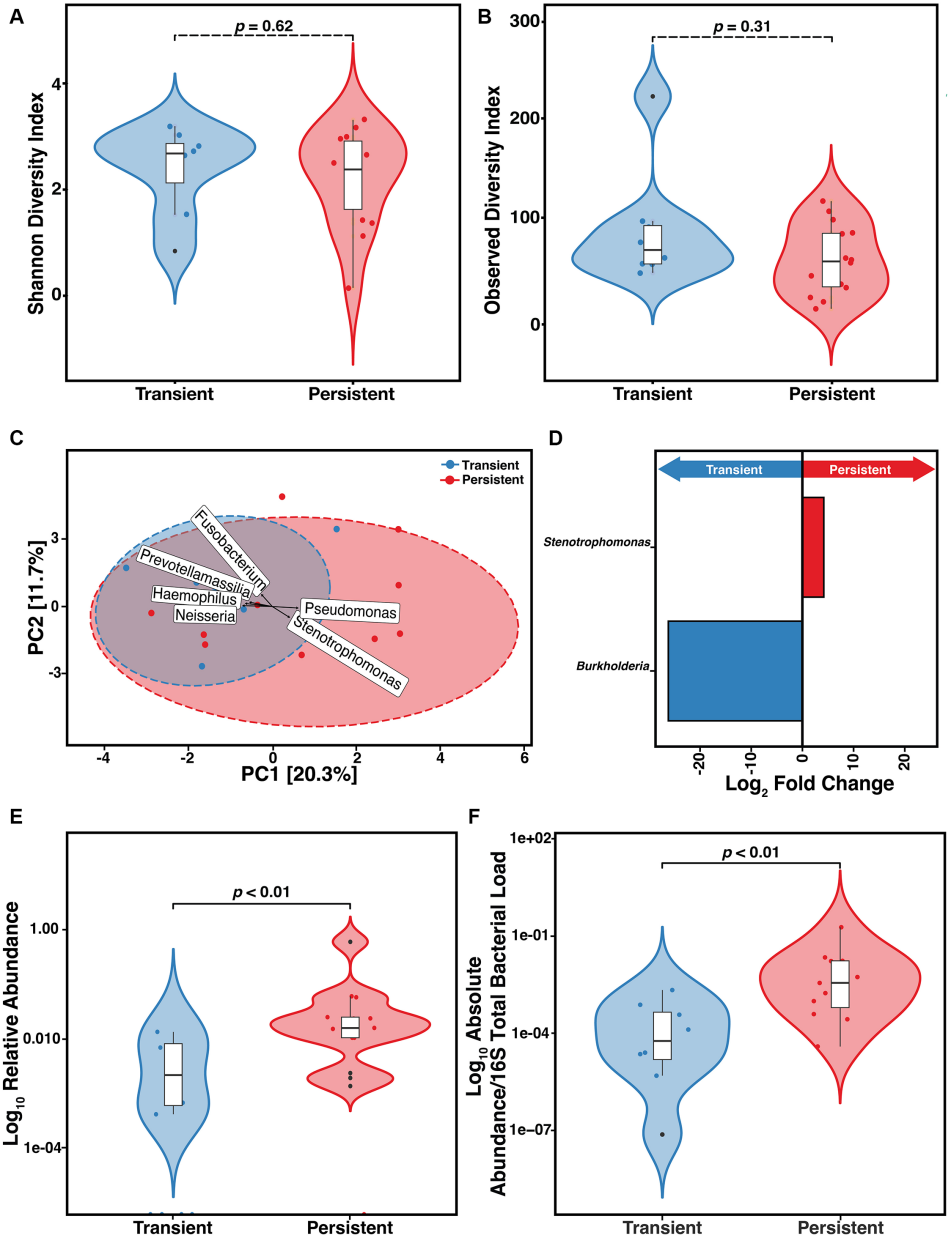
Risk of developing persistent (*n* = 14) versus transient (*n* = 8) *S. maltophilia* infection in CF. **(A)** Shannon Diversity Index (SDI) (Wilcoxon, *p* = 0.62) **(B)** Observed Diversity Index (ODI) (Wilcoxon, *p* = 0.31). **(C)** Principal Component Analysis (PCA) with center-log ratio (CLR) transformation; (PERMANOVA, *p* = 0.03) and with top 6 taxa, present in ≥10% of samples, influencing unconstrained PCA clustering. **(D)** Taxa with significant log fold changes in relative abundance between transient and persistent infections, detected by DESeq2. **(E)** Relative abundance of *S. maltophilia* for transient and persistent at-infection case samples presented in the Log10 scale (Wilcoxon, *p* = 0.0044). **(F)** Absolute abundance/total 16S bacterial load for transient (*n* = 8) and persistent (*n* = 11) at-infection case samples presented in the Log10 scale (Wilcoxon, *p* = 0.005). Data points are represented on violin plots as colored dots. Outlier data points are black and are defined as any point that were beyond 1.5 times the interquartile range.

We found that of the 33 at-infection samples, 18 (54.5%) were derived from outcomes that were classified as persistent. We assessed if the constituents of the microbiome differed between those with persistent versus transient infection. We did not find any difference in the α-diversity as measured by either SDI (*p* = 0.62) or ODI (*p* = 0.31) (Figures 4A,B). To determine if sputum from those with transient and persistent infections clustered together or separately, PCA was compared (Figure 4C). We found a significant difference in the clustering based on the Aitchison distance of the transient and persistent At samples (PERMANOVA, *F* = 1.7, R^2^ = 7.8%, *p* = 0.03). However, these results were not supported when the data were stratified by participant ID (see Supplementary File). The top 6 taxa that contributed to PCA clustering dissimilarity between infection outcome were determined. DESeq2 was used to establish the DAA of the ASVs that differed in relative abundance in samples collected at-infection based on eventual outcomes (Heirali et al., 2019; Cuthbertson et al., 2021). We found that *Pseudomonas* and *Stenotrophomonas* correlated with the difference in clustering in the persistent At samples whereas *Fusobacterium, Neisseria, Prevotellamassilia*, and *Haemophilus* were associated with the clustering difference in the transient group (Figure 4C). According to the DESeq2 data, only *Stenotrophomonas* (*p* = 0.01) was significantly enriched at incident infection of those with persistent infection and *Burkholderia* (*p* = 8.8e-14) was enriched in those with transient infection (Figure 4D). We assessed if the difference in the relative abundance of *Stenotrophomonas* at incident infection correlated with outcome. We found those who ultimately developed persistent infection had higher *S. maltophilia* relative abundance than those with transient infection (Figure 4E) (Wilcoxon, *p* = 0.0043). This relative abundance was confirmed via qPCR establishing the absolute ratio of *S. maltophilia* to total 16S total bacterial load (Figure 4F) (Wilcoxon, *p* = 0.005). Those who ultimately progressed to persistent infection had higher absolute (normalized) bioburden of *S. maltophilia* at incident infection than those who were merely transiently infected.

## 4 Discussion

Infection with *S. maltophilia* in pwCF has been associated with increased morbidity and mortality (Gajdács and Urbán, 2019). Risk factors among pwCF for *S. maltophilia* infection have been identified and include those with increased lung function decline (Stanojevic et al., 2013), and antibiotic treatments (Denton et al., 1996), specifically antibiotics used to treat *Pseudomonas aeruginosa* infections such as tobramycin (Burns et al., 1999). However, the risk of infection acquisition or persistence leading to chronic infection has not been widely explored. Research on the natural history of incident *S. maltophilia* infection and its association with the sputum microbiome may serve to fill this knowledge gap by helping to understand the changes in microbial community structure that may support or hinder the invasion and/or persistence of *S. maltophilia* infections.

The CF airway microbiome is known to host a diverse community of microorganisms that have been established as relatively stable within patients and so may serve as a patient-specific tool for prognostication. Recent work using animal models found that the microbial communities within the lung can alter infection dynamics – owing to the relationship between the microbiome and airway infections (Duan et al., 2003; Stacy et al., 2016; McDaniel et al., 2020). Others have sought to identify the role of the CF airway microbiome as a risk factor for infection with other CF pathogens such as *Nontuberculous mycobacteria* (Breen et al., 2022).

We first sought to profile the CF lower airway microbiome of pwCF who acquired incident *S. maltophilia* infections using 16S allelic sequencing. This commonly used technique facilitated detailed exploration of the bacterial community of the CF respiratory microbiome (Cuthbertson et al., 2020; Hahn et al., 2020; Deschamp et al., 2023) providing the means for a thorough analysis of the microbial community, including very rare constituents. After evaluating variations in community structure throughout the natural history of infection, we found no significant differences in microbial diversity—neither within nor between individuals with cystic fibrosis (pwCF)—nor in the community structure during the natural progression of *S. maltophilia* infection. *Stenotrophomonas* ASV did not associate with any other specific ASV suggesting this pathogen exists independently of other species. This expands on previously published work that has established the stability of the intra-diversity of the airway microbiome in pwCF (Fodor et al., 2012). This also supports the notion that individuals have a unique community structure that is relatively unperturbed by incident infection with *S. maltophilia*. We did observe significant differences in both the relative abundance and absolute abundance of *S. maltophilia* relative to the absolute abundance of total 16S rRNA (representing the total bacterial bioburden) between the Pre and At stages, as well as between the At and Post stages. This outcome was anticipated, given our expectation that *S. maltophilia* would be less abundant before the onset of infection, more abundant at the onset, and then less abundant post-infection.

To understand the microbiome’s role in *S. maltophilia* infection we used a matched cohort study design. We did not find any significant differences in baseline demographics nor markers of clinical disease between pwCF experiencing *S. maltophilia* incident infection and the uninfected controls in our relatively modest cohort. We did find a significant difference in community structure as measured by both the SDI α-diversity and community clustering. We observed a significant increase in the ASVs corresponding to *Stenotrophomonas* in sputum collected prior to its first identification by cultured growth (i.e., these samples were culture negative, subsequent samples were culture positive) relative to controls. This may be due to the increased sensitivity of nucleic acid testing compared to culture data. This has been well-established in incident infections with *P. aeruginosa* in pwCF (Deschaght et al., 2009, 2010), and more recently, *S. maltophilia* (Rocchetti et al., 2018). In fact, an increasing body of evidence suggests that the microbial community analysis provides a more comprehensive assessment of classical CF pathogens’ role in CF airways than culture alone – including identifying the risk of disproportionate disease progression (Acosta et al., 2018), and treatment response (Hansen, 2012; Waters et al., 2013; Berdah et al., 2018; Mojica et al., 2022). This evidence underscores the advantage of nucleic acid testing and highlights the importance of early detection.

The secondary outcome of this work was to determine if there was an association between the CF lower airway microbiome composition at incident *S. maltophilia* infection and risk of persistence. Routine clinical parameters and demographics did not differentiate these two groups. We did not observe differences in α-diversity between cohorts at initial infection. This was somewhat unexpected given *S. maltophilia*’s association with clinical decline and previous studies demonstrating that pwCF with decreasing lung function have stable, but less diverse microbiomes (Goss et al., 2004; Metzger et al., 2021). However, we did observe clustering between samples as represented by the β-diversity that were associated with infection outcome. We found *Pseudomonas*, and *Stenotrophomonas* were drivers of the difference in samples from subjects that developed persistent infection and *Fusobacterium, Neisseria, Prevotellamassilia*, and *Haemophilus* drove the difference in clustering within the samples from those experiencing only transient infection. We then investigated the ASVs that differed in sputum collected at the actual time of incident infection between those that ultimately developed persistent versus transient infections. We found that the relative abundance of *Stenotrophomonas* was enriched in the persistent infection samples and the relative abundance of *Burkholderia* was enriched in the transient infection samples. We confirmed the relative enrichment of *S. maltophilia* using qPCR. This observation may suggest that the initial bioburden of incident infections can influence the outcome of infections, which may justify early antimicrobial intervention to reduce the risk of progression to long-term infection, as has become the standard of care with *P. aeruginosa* early eradication (Casaredi et al., 2022).

While the participants in our study were not receiving highly effective modulator treatments (HEMT), it is important to recognize the profound changes in HEMT use in the last several years and its impact on the CF airway microbiome in future directions of this work. HEMT has been associated with several important changes to the airway microbiome including a more even distribution of individual bacterial species, a decrease in bacterial load, and a shift from diverse individual microbial communities to more similar communities dominated by typically healthy airway inhabitants (Man et al., 2017; Pallenberg et al., 2022). However, long-term impacts of HEMT on the airway microbiome are still largely unknown. Future considerations for both short-term and long-term impacts of HEMT on microbial composition and incident infection acquisition and persistence will need further investigation.

Several notable limitations to this study exist and require consideration. Firstly, this is a single-center, retrospective study, which limits the scalability of the results. Furthermore, there are several confounding variables that required consideration. To account for these confounding variables, we developed a cohort of matched participants, reported their demographics and characteristics, and excluded sputum samples that were collected during new antimicrobial treatments. Our inclusion/exclusion criteria reduced the potential sample size, which lowered the overall power of the study while ensuring homogeneity. However, the results acquired from the species accumulation curve showed an appropriate sequencing depth, which suggests that the sample size adequately represents our expected observed diversity. This study assessed the respiratory microbiome in pwCF at Pre, At and Post incident *S. maltophilia* infection. However, it is important to note that the study did not capture the modest daily dynamic changes in the respiratory microbiome that have been documented to occur within individuals (Caverly et al., 2019). Instead, this study examined patterns observed around the occurrence of incident *S. maltophilia* infection. The retrospective nature of this study limits our ability to generate any causal relationships among the data. While many studies have justified that the use of sputum accurately represents the lower airway microbiome (Hogan et al., 2016; Zemanick et al., 2017; Lu et al., 2020), there is of course the fact that some degree of contamination from the oral flora occurs as sputum passes through the oral cavity before expectoration and sample collection. However, clinicians do not require that treatment models accurately capture all aspects of the *in-situ* situation (i.e., susceptibility testing is not reflective of the environment CF pathogens live), but rather require tools that better predict clinical outcomes. Indeed, there is evidence that microbial community analyses may correlate with clinical outcomes better than simple culture presence/absence (Acosta et al. 2018).

## 5 Conclusion

Herein we observed that the CF sputum microbiome – while not altered by incident infection with *S. maltophilia* – may be associated with infection acquisition and persistence risk. We further established that molecular tools are more sensitive for pathogen identification than classical culture-based methods, even for *S. maltophilia*. Additional research is needed to establish whether these observed differences in bacterial abundance contribute to the risk of either acquiring incident infections or developing persistent infections with *S. maltophilia*. Here, we have highlighted the potential of CF sputum microbiome analysis as a future clinical tool to improve infection diagnosis and management in pwCF.

## Data availability statement

The data presented in the study are deposited in the NCBI BioSample database repository, accession number PRJNA1033679.

## Ethics statement

The studies involving humans were approved by University of Calgary Conjoint Health Region Ethics Board. The studies were conducted in accordance with the local legislation and institutional requirements. The participants provided their written informed consent to participate in this study.

## Author contributions

LAB: Data curation, Formal analysis, Investigation, Methodology, Writing – original draft. NA: Data curation, Formal analysis, Methodology, Project administration, Supervision, Writing – review & editing. CT: Formal analysis, Project administration, Validation, Writing – review & editing. JC: Investigation, Writing – review & editing. BW: Formal analysis, Investigation, Project administration, Supervision, Writing – review & editing. LB: Investigation, Methodology, Writing – review & editing. KE: Supervision, Validation, Writing – review & editing. DC-M: Validation, Writing – review & editing. JMC: Funding acquisition, Investigation, Project administration, Supervision, Writing – review & editing. HR: Data curation, Project administration, Writing – review & editing. MS: Data curation, Funding acquisition, Investigation, Methodology, Supervision, Writing – review & editing. MP: Conceptualization, Formal analysis, Funding acquisition, Investigation, Project administration, Resources, Supervision, Validation, Writing – review & editing.
