# Supplementary material for: The airway microbiome of persons with cystic fibrosis correlates with acquisition and microbiological outcomes of incident Stenotrophomonas maltophilia infection
Source: Front Microbiol. 2024 Apr 16;15:1353145. doi: 10.3389/fmicb.2024.1353145 (PMC11059027; doi:10.3389/fmicb.2024.1353145)
Supplement: Supplementary file 1 [file Data_Sheet_1.docx]

Supplementary Material

# Methods

## Clinical Information Collected

Clinical data on cases and controls comprised age, sex, height, weight, co-morbidities, CFTR mutation genotype (including functional classes encompassing Class I-VI), infection history, medications, and treatments (including initiation of modulator therapies) were established at the time of incident infection. Pulmonary function values with percent predicted forced expiratory volume in one second (ppFEV_1_) and percent predicted forced vital capacity (ppFVC), at the time of each sample collection, although cohort characteristics are described at incident infection.

## Clinical Outcomes Definitions

Definitions of chronic infection for *S. maltophilia* have not been validated, and *S. maltophilia* infection does not commonly progress to chronic infection as is observed with *Pseudomonas aeruginosa* (Brooke 2012),We considered persistent infections as cases where subjects with an *S. maltophilia* infection had at least half of their cultures positive for infection following the incident infection sample with a minimum of three samples within the year following initial infection. Transient infection as an outcome was defined when subjects had less than half of their sputum cultures were positive for *S. maltophilia* within the year following their incident infection date where a minimum of 3 sputum samples were obtained.

## DNA Extraction

Select sputum was taken from the -80°C storage freezers and allowed to thaw on ice. Once thawed, each sample was then homogenized using a sterile 1ml tuberculin tip syringe with 18-gauge needle. A volume of 300μL of the homogenized sample was then mixed with 800μL of 200mM NaPO_4_ (pH 8) and 100μL of GES buffer (0.5mmol guanidine thiocyanate, 0.01mmol, ethylenediaminetetraacetic acid (EDTA), 0.003mmol N-lauroyl sarcosine). The samples were then mechanically lysed with 0.2g of 0.1mm glass beads (Mo Bio Laboratories, #13118- 50) in the PowerLyzer set to homogenize at 3000rpm for 3 minutes. Enzymatic lysis was conducted using 50μL of 100mg/mL lysozyme (Sigma-Aldrich, #L6876-10G) and 10μL of 10mg/mL RNase A (Qiagen, #19101) with an initial incubation at 37°C for 90 minutes followed by the addition of 25μL of 25% SDS, 25μL Proteinase K enzymatic solution (0.05M proteinase K, 0.015M Tris, 0.015M calcium acetate) (Sigma-Aldrich, #P2308-1G), and 62.5μL 5M NaCl with a final incubation at 65°C for another 90 minutes to separate the sample from any cellular proteins. The samples were then centrifuged at 13,500g for 5 minutes to separate the sample from beads and any other unwanted cellular material. DNA extraction was conducted by adding 900μL supernatant to 900μL 25:24:1 phenol-chloroform-isoamyl alcohol, mixing well by vortexing for 20 seconds and then centrifuging at 13000g for 10 minutes. A volume of 600μL of the supernatant from the centrifuged sample was mixed with 600μL of DNA binding buffer and spun through a DNA column (DNA Clean and Concentrator-25, Zymo, #D4034) at 12,000g for 1 minute. A total of 400μL of wash buffer (DNA Clean and Concentrator-25, Zymo, #D4034) was added to each column and spun at 12,000g for 1 minute. The extracted DNA was then eluted from the DNA column using 50μL of ultrapure double-distilled H_2_O (ddH_2_O) pre-heated to 65°C. The extracted DNA samples were quantified using a Nanodrop-1000 (Thermo Scientific, Waltham, MA) to determine the amount of DNA present in the sample in ng/μL and stored at 20°C. The amount of DNA present was standardized to 100ng prior to PCR amplification.

## 16S rRNA Amplification and Sequencing

The V3-V4 region was chosen because it has been shown to allow for improved identification of taxa as well as increased diversity (Bartram et al. 2011).Moreover, as the cost of sequencing has become more affordable, sequencing longer reads has become easier allowing for improved microbiome analysis.

Three polymerase chain reactions (PCR) were carried out for each sample – the total reaction mixture was aliquoted equally (16.6μL) into three separate PCR tubes to reduce concatenated products. Each reaction contained 25.25μL ultra-pure H_2_O (Invitrogen, Waltham, Massachusetts, USA), 5μL 10X buffer (Invitrogen) 1.5μL 50 mM MgCl_2_, 1μL 10mM concentration of each deoxynuceoside triphosphate (dNTP) ( Invitrogen), 1μL 1μM V3F primer, 10μL V4Rmod2 primer, 100ng of sample template, 1μL of 20mg/mL bovine serum, albumin (BSA) (New England BioLabs, Ipswich, Massachusetts, USA), and 0.25μL of *Taq* polymerase (Invitrogen). The PCR cycle conditions included an initial denaturation step at 94°C for 5 minutes followed by 30 cycles of: 94°C for 1 minute, 47°C for 30 seconds, 72°C for 40 seconds. Followed with will a final single-time extension step at 72°C for 10 minutes in a Bio-Rad thermocycler (Bio-Res, Mississauga, Ontario).

The PCR products were analyzed by gel electrophoresis on a 1% agarose gel. Any samples with bands outside of the 590bp region were re-processed at a higher sample concentration and the bands of the correct size recovered using a QIAquick gel extraction kit (Qiagen, Mississauga, Ontario).

Reagent blanks were included for each set of DNA extractions and excluded and repeated as necessary if the samples contained PCR products.

## Sequence Processing

Sequence processing was conducted in R Studio (v1.4). The barcoded primers and adaptor sequences were removed using Cutadapt (v1.2.1) (Martin 2011),followed by filtering trimming, sample inference, alignment, and finally, taxonomy assignment using the Divisive Amplicon Denoising Algorithm (DADA) 2 pipeline, an open source R package (Callahan et al. 2016). Sample inference was based on error models constructed for the amplicon dataset. Aligned sequences were inferred as exact amplicon sequence variants (ASVs) from the amplicon sequencing data. The use of ASVs as the unit of marker-gene analysis was used over Operational Taxonomic Units (OTUs) for improved resolution since ASVs can be resolved down to single-nucleotide differences over the sequenced region (Callahan et al. 2016). Taxonomy assignment was conducted using the assignTaxonomy function from the DADA2 package, which provides a native implementation of the naïve Bayesian classifier method and uses a training set of reference sequences with known taxonomy to provide an output of taxonomic assignments with at least minBoot bootstrap confidence (Callahan et al. 2016). The reference database used in this study utilised the RDP trainset 18 (v11.5) for taxonomic assignment (Callahan et al. 2016). Agglomeration using the tax glom function from the Phyloseq R package was used to account for instances of multiple ASV assignments to the same genus (Callahan et al. 2016). This reduced the number of ASVs while retaining the same genus-level assignments. Species accumulation curves (SAC) were obtained with the specaccum function of the vegan R package.

## Microbial Communities Analysis

Ratio transformation using the CLR method was used to make the data symmetric and linearly related allowing the log-ratio abundances of features relative to other features (Gloor et al. 2017)A second permutational analysis was performed where permutations were stratified to participant ID to assess the random effects of having multiple pre-infection measures per participant on the permutational analysis.

## Strain-Level Identification

Pulse-field gel electrophoresis (PFGE) is a method of genotyping bacterial strains. PFGE has been considered the gold-standard for bacterial strain typing (Parkins et al. 2014)Furthermore, PFGE has been used extensively in microbiome profiling (Dos Santos *et al.*, 2021). To conduct PFGE analysis, bacterial isolates from the sputum samples were grown on fresh TSY agar and cultivated at 37°C, overnight. The grown colonies were suspended in 3mL of suspension buffer (1M NaCl, 10mM Tris pH 7.6) and mixed 1 to 1 with molten 1% seakem gold agarose (heated until melting temperature) and then poured into a plug mold and allowed to cool. The plug containing the suspended colonies within the agar was then subjected to chemical and enzymatic lysis with lysis buffer (1M NaCl, 100mM EDTA [pH 7.5], 0.5% Brij-58, 0.5% Sarcosyl, 0.2% Deoxycholate, 6mM Tris-HCl [pH 7.6], 1mg/ml Lysozyme, 20μg/mL RNase) for 4h 37^o^C followed by EPS buffer (0.5M EDTA [pH 9 − 9.5], 1% Sarcosyl, 70μg/mL Proteinase K ) overnight, 50°C to lyse, remove proteins and release the bacterial DNA. The DNA was then washed 3x in 10mM Tris, 0.1mMEDTA and digested using 20U Spe1 restriction enzyme for 4h at 37°C to cut into specific strain patterns. The agarose plug containing the DNA were ran on a 1% seakem gold agarose gel in 10mM Tris, 0.1mM EDTA and was subjected to electrophoresis on a Chef Mapper in 0.5x TBE buffer 10°C, 6V, 120°C, 5sec initial switch, 45sec final switch for 20h The gel was stained with Gel-red for imaging and analyzed using Bionumerics (Applied Math) using UPGMA, DICE analysis at 1.5% optimization and 2% tolerance with the cluster analysis method, unweighted pair-groups method using arithmetic averages (UPGMA) (Hahm et al. 2003)The DICE coefficient was obtained by comparing bands at specific positions and uses probability theory to determine whether the bands were distinct (Dice 1945).

## Absolute Quantification of *S. maltophilia*

A standard curve was created using serial dilutions from a known concentration of DNA from a 500ng gBlock. The gBlock standards were spun down to concentrate the pellet and to ensure that all the sample was collected in the bottom of the tube before resuspension. A volume of 50μL of sterile ddH_2_O was added to the tube to create a concentration of 2.5$\text{×}\text{10}^{\text{10}}$copies/μL. The gBlock was then diluted to a concentration of $\text{1×}\text{10}^{\text{8}}$copies/μL. The standard curve will be created by adding 5μL of sterile ddH_2_O and repeated 8 times to create serial 10-fold dilutions (ranging from $\text{8×}\text{10}^{\text{8}}$copies/μL to 8 copies/μL). The dilutions of these standards were verified using a Nanodrop-1000 Spectrophotometer. Each qPCR was completed in triplicate for each sample to ensure accuracy. The DNA corresponding to the selected primers were amplified and measured by the fluorescence of the probe once bound to the dsDNA in each PCR cycle – known as the quantification cycle (C_q_). The C_q_ value of the sample was measured against the C_q_ of the standard curve to determine the presence and quantity of the pathogen present in each sample. The 16S region specific to *S. maltophilia* was targeted. The primer, probe, and protocol used to amplify *S. maltophilia* was adapted from the work published by Fraser et al. 2019 (Fraser et al. 2019).

**1.9 *S. maltophilia* Susceptibility Testing**

Antibiotic susceptibility testing was performed in real-time to trimethoprim-sulfamethoxazole using the Kirby-Bauer disc method as per the Clinical and Laboratory Standards Institute (CLSI) (Wayne 2009)(ref).

# Results

## Differences in the Microbiome Amongst pwCF who Acquire *S. maltophilia* Infection

Due to limited residual DNA, we were unable to conduct qPCR analysis on 6 pre-infection sputum samples and 7 control sputum samples.

We assessed the correlation between the relative abundance data and the absolute abundance data of *S. maltophilia* and found a statistically significant positive correlation (Spearman’s rank correlation, *p*<$\text{2.2}\text{e}^{\text{-16}}$).

## Microbiome Structure at Incident Acquisition as a Function of Infection Outcome

When stratified by participant ID, no significant difference in the clustering of the transient and persistent At samples was observed (PERMANOVA, F=0.7, R2=3.4%, *p*=1.00).

Due to a lack of available sample, we were unable to conduct qPCR analysis on 3 persistent at-infection case samples.

We assessed the correlation between the relative abundance data and the absolute abundance data of *S. maltophilia* and found a statistically significant positive correlation (Spearman’s rank correlation, *p*=4.7*e*^-10^).

**Supplementary Figure 1**. Species accumulation curve to assess the quality of sequencing.

**Supplementary Figure 2.** Pulse-field gel electrophoresis (PFGE) data for strain-level identification of cases where multiple episodes of *S. maltophilia* infection were recorded. A total of 6 cases had multiple infections with *S. maltophilia* whose cultured isolates were identified to be different by PFGE.

**
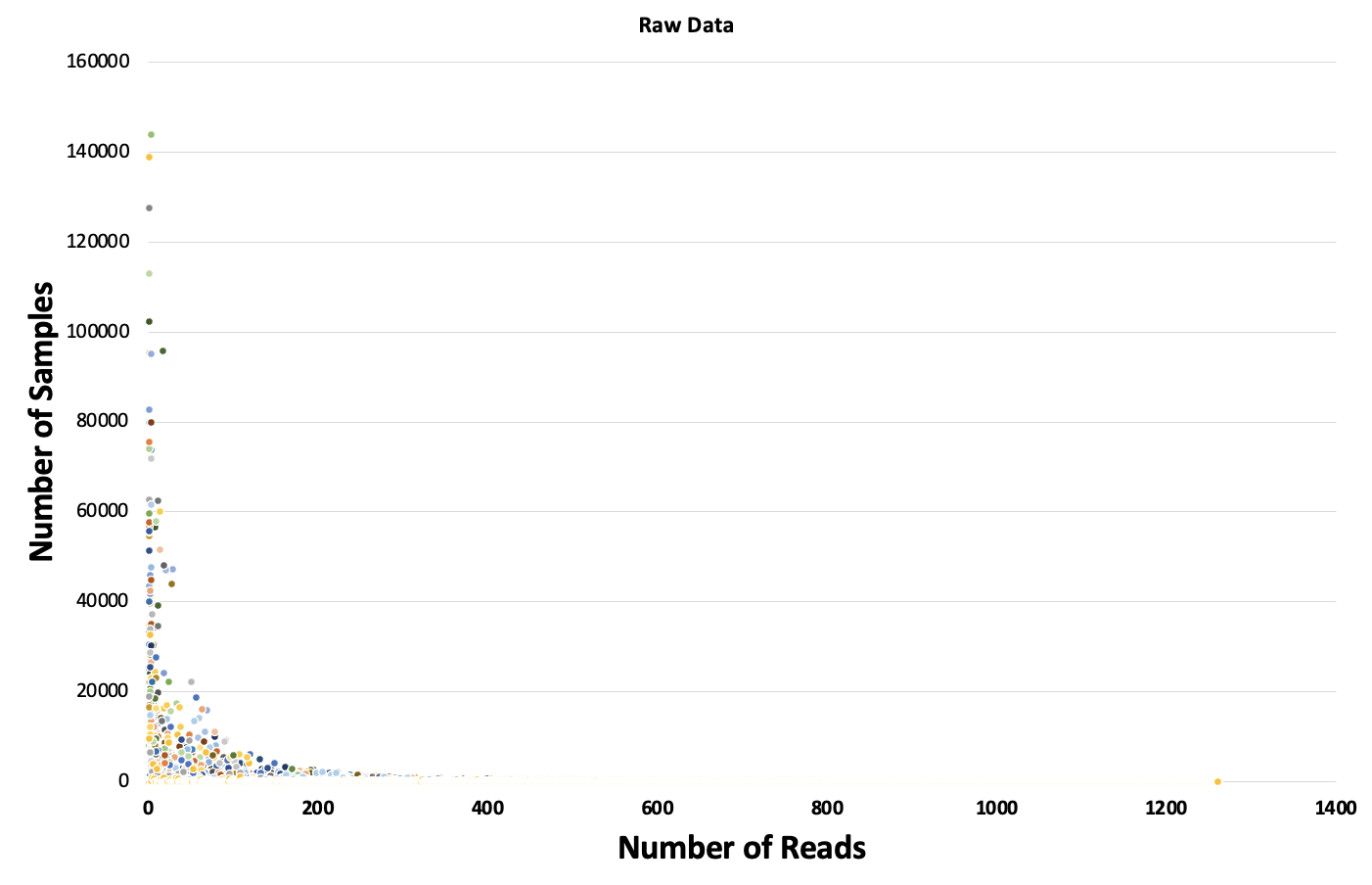
**

**Supplementary Figure 3.** Distribution of the number of samples across the number reads of the raw sequencing data. The distribution follows a Poisson log-normal distribution (p<0.0001; Chi-square goodness of fit).

**Supplementary Table 1.** Contingency table for trimethoprim-sulfamethoxazole (TMP-SMZ) susceptibility of *S. maltophilia* strains cultured from patients with either transient or persistent infections. A total of 20 patients (n=13 transient; n=7 persistent) had susceptibility testing completed at the time of *S. maltophilia* infection. Susceptibility data was unavailable for 13 samples (8 transient and 5 persistent).

|  | **Resistant** | **Susceptible** |
| --- | --- | --- |
| **Transient** | 0 | 7 |
| **Persistent** | 0 | 13 |

**References**

Bartram, Andrea K., Michael D. J. Lynch, Jennifer C. Stearns, Gabriel Moreno-Hagelsieb, and Josh D. Neufeld. 2011. ‘Generation of Multimillion-Sequence 16S rRNA Gene Libraries from Complex Microbial Communities by Assembling Paired-End Illumina Reads’. *Applied and Environmental Microbiology* 77 (11): 3846–52. https://doi.org/10.1128/AEM.02772-10.

Brooke, Joanna S. 2012. ‘Stenotrophomonas Maltophilia: An Emerging Global Opportunistic Pathogen’. *Clinical Microbiology Reviews* 25 (1): 2–41. https://doi.org/10.1128/CMR.00019-11.

Callahan, Benjamin J, Paul J McMurdie, Michael J Rosen, Andrew W Han, Amy Jo A Johnson, and Susan P Holmes. 2016. ‘DADA2: High-Resolution Sample Inference from Illumina Amplicon Data’. *Nature Methods* 13 (7): 581–83. https://doi.org/10.1038/nmeth.3869.

Dice, Lee R. 1945. ‘Measures of the Amount of Ecologic Association Between Species’. *Ecology* 26 (3): 297–302. https://doi.org/10.2307/1932409.

Fraser, Tamieka A., Mikaela G. Bell, Patrick N.A. Harris, Scott C. Bell, Haakon Bergh, Thuy-Khanh Nguyen, Timothy J. Kidd, Graeme R. Nimmo, Derek S. Sarovich, and Erin P. Price. 2019. ‘Quantitative Real-Time PCR Assay for the Rapid Identification of the Multidrug-Resistant Bacterial Pathogen *Stenotrophomonas Maltophilia*’. Preprint. Microbiology. https://doi.org/10.1101/702985.

Gloor, Gregory B., Jean M. Macklaim, Vera Pawlowsky-Glahn, and Juan J. Egozcue. 2017. ‘Microbiome Datasets Are Compositional: And This Is Not Optional’. *Frontiers in Microbiology* 8 (November): 2224. https://doi.org/10.3389/fmicb.2017.02224.

Hahm, Byoung-Kwon, Yadilka Maldonado, Edgar Schreiber, Arun K. Bhunia, and Cindy H. Nakatsu. 2003. ‘Subtyping of Foodborne and Environmental Isolates of Escherichia Coli by Multiplex-PCR, Rep-PCR, PFGE, Ribotyping and AFLP’. *Journal of Microbiological Methods* 53 (3): 387–99. https://doi.org/10.1016/S0167-7012(02)00259-2.

Martin, Marcel. 2011. ‘Cutadapt Removes Adapter Sequences from High-Throughput Sequencing Reads’. *EMBnet.Journal* 17 (1): 10. https://doi.org/10.14806/ej.17.1.200.

Parkins, Michael D., Bryan A. Glezerson, Christopher D. Sibley, Kristen A. Sibley, Jessica Duong, Swathi Purighalla, Christopher H. Mody, et al. 2014. ‘Twenty-Five-Year Outbreak of Pseudomonas Aeruginosa Infecting Individuals with Cystic Fibrosis: Identification of the Prairie Epidemic Strain’. Edited by P. H. Gilligan. *Journal of Clinical Microbiology* 52 (4): 1127–35. https://doi.org/10.1128/JCM.03218-13.

Wayne, PA. 2009. ‘Performance Standards for Antimicrobial Disk Susceptibility Tests’. CLSI document M02-A10.
